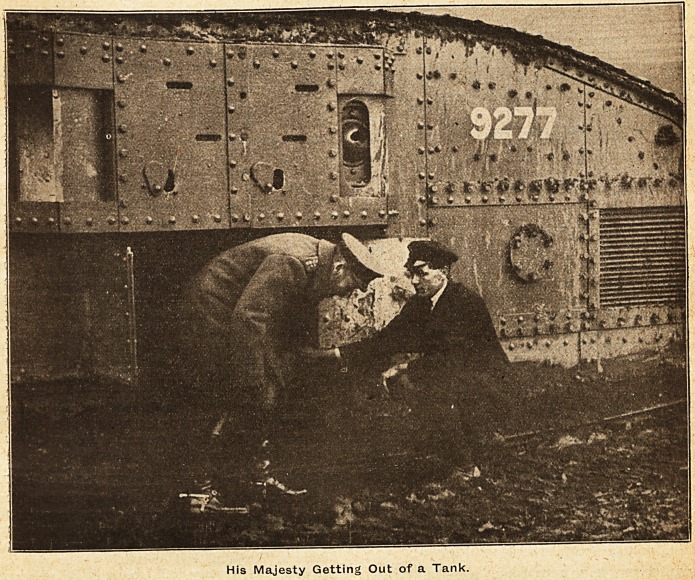# H.M. King George at Lincoln

**Published:** 1918-04-20

**Authors:** 


					April 20, 1918. THE HOSPITAL 43
H.M. KING GEORGE AT LINCOLN.
His Majesty Getting Out of a Tank.

				

## Figures and Tables

**Figure f1:**